# Emerging roles for multifunctional ion channel auxiliary subunits in cancer

**DOI:** 10.1016/j.ceca.2019.04.005

**Published:** 2019-06

**Authors:** Alexander S. Haworth, William J. Brackenbury

**Affiliations:** a Department of Biology, University of York, Heslington, York, YO10 5DD, UK; b York Biomedical Research Institute, University of York, Heslington, York, YO10 5DD, UK

**Keywords:** BK, large-conductance calcium-activated potassium channel, CaCC, calcium-activated chloride channel, CAM, cell-adhesion molecule, CLC, voltage-gated chloride channel, CLCA, chloride channel accessory, DREAM, downstream regulatory element antagonistic modulator, GIRK, G-protein inwardly rectifying potassium channel, KChIP, potassium channel interacting protein, Kir, inwardly-rectifying potassium channel, SUR, sulfonylurea receptor, VGCC, voltage-gated calcium channel, VGKC, voltage-gated potassium channel, VGSC, voltage-gated sodium channel, Auxiliary subunit, Cancer, Calcium channel, Chloride channel, Potassium channel, Sodium channel

## Abstract

Several superfamilies of plasma membrane channels which regulate transmembrane ion flux have also been shown to regulate a multitude of cellular processes, including proliferation and migration. Ion channels are typically multimeric complexes consisting of conducting subunits and auxiliary, non-conducting subunits. Auxiliary subunits modulate the function of conducting subunits and have putative non-conducting roles, further expanding the repertoire of cellular processes governed by ion channel complexes to processes such as transcellular adhesion and gene transcription. Given this expansive influence of ion channels on cellular behaviour it is perhaps no surprise that aberrant ion channel expression is a common occurrence in cancer. This review will focus on the conducting and non-conducting roles of the auxiliary subunits of various Ca^2+^, K^+^, Na^+^ and Cl^−^ channels and the burgeoning evidence linking such auxiliary subunits to cancer. Several subunits are upregulated (e.g. Ca_v_β, Ca_v_γ) and downregulated (e.g. K_v_β) in cancer, while other subunits have been functionally implicated as oncogenes (e.g. Na_v_β_1_, Ca_v_α_2_*δ*_1_) and tumour suppressor genes (e.g. CLCA2, KCNE2, BKγ_1_) based on *in vivo* studies. The strengthening link between ion channel auxiliary subunits and cancer has exposed these subunits as potential biomarkers and therapeutic targets. However further mechanistic understanding is required into how these subunits contribute to tumour progression before their therapeutic potential can be fully realised.

## Highlights

• Ion channels consist of conducting and non-conducting (auxiliary) subunits.
• Auxiliary subunits regulate ion conductance and have non-conducting roles.
• Ion channels control diverse cellular processes and are aberrantly expressed in cancer.
• Auxiliary subunits play major roles in cancer cells, including regulating adhesion, migration, invasion and gene expression.

## Graphical abstract

**Figure:**
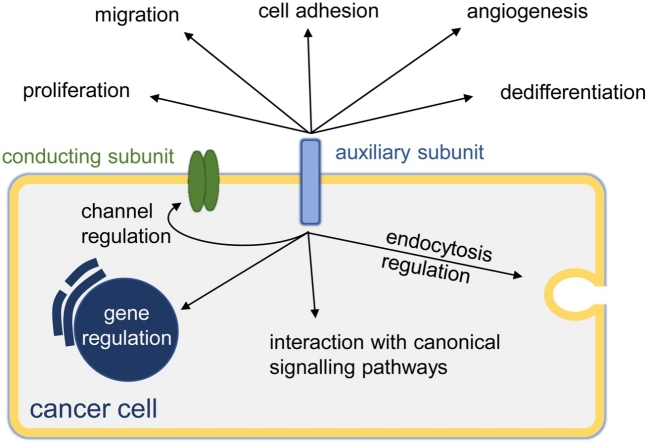

## 1 Introduction

Ion channels are heteromeric membrane protein complexes which permit transmembrane ion conduction. Several ion channels, e.g. K^+^ channels and voltage-gated Na^+^ channels (VGSCs), are notable for regulating membrane potential in excitable cells [1], but an expanding repertoire of other cellular processes, such as proliferation, differentiation [2], cell volume control and migration [3,4], are also known to be influenced by ion channels. Owing to their extensive impact on cellular function, it is no surprise that ion channel dysregulation is a common characteristic in cancer [5]. Ion channels are often multimeric, with ion-conducting subunits accompanied by non-conducting auxiliary subunits [6]. Auxiliary subunit-mediated modulation of the conducting subunit is well established but increasing evidence has unveiled a multitude of non-conducting roles for these proteins as well [[7], [8], [9], [10], [11], [12], [13], [14]]. An emerging field has focused on investigating auxiliary subunits in cancer, which, like the conducting subunits, are often aberrantly expressed and could represent novel therapeutic targets. In this review, we dissect the conducting and non-conducting roles of the auxiliary subunits of Ca^2+^, K^+^, Na^+^ and Cl^−^ channels and the growing evidence supporting a link to cancer.

## 2 Ca^2+^ channels

**Fig. 1:**
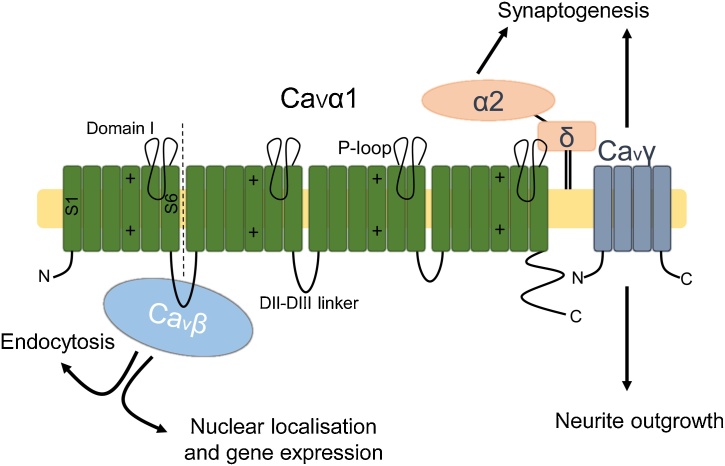
Voltage-gated Ca^2+^ channel auxiliary subunits. Voltage-gated Ca^2+^ channels (VGCCs) are composed of a conducting α1 subunit accompanied and functionally modulated by Ca_v_β, α_2_δ and Ca_v_γ subunits [20]. α1 consists of four domains (domains I-IV), each consisting of six segments (S1-S6). The voltage-sensing domain is found within S4 of each domain and the pore consists of the P-loop found between S5-6 of each domain. Ca_v_β modulates Ca^2+^ influx via binding the DI-DII linker of α1. Ca_v_βs are also involved in regulating gene expression and endocytosis [22,[36], [37], [38],^40^,^44^]. α_2_δ subunits are extracellular proteins that remain associated to the membrane via a GPI-anchor [54]. α_2_δ subunits are involved in synaptogenesis [65]. Ca_v_γ subunits are four-pass transmembrane proteins also involved in cervical ganglion neurite outgrowth and synaptogenesis [108,109].

Ca^2+^ channels regulate a multitude of cellular processes; accordingly, much research has focused on various Ca^2+^ channels in cancer, including voltage-gated Ca^2+^ channels (VGCCs) [15], STIM and Orai [16], and TRP channels [17]. In terms of Ca^2+^ channel auxiliary subunits however, only VGCC auxiliary subunits have received notable attention thus far. VGCCs are transmembrane complexes responsible for the inward Ca^2+^ current seen in excitable cells following depolarisation, however VGCCs are also expressed in other non-excitable cell types, e.g. osteoblasts and osteoclasts [18,19]. VGCCs are composed of a Ca^2+^-conducting α_1_ subunit (Ca_v_1-3.*x*) associated with multiple auxiliary subunits (α_2_δ_1-4_, β_1-4_, γ_1-8_), with the exception of Ca_v_3.*x*, which can form a T-type Ca^2+^ channel in the absence of an associated auxiliary subunit (Fig. 1) [20]. A Ca_v_1/2 subunit is joined at the membrane by an α_2_δ-, β-, and potentially a γ-subunit, although γ-subunits are not always precipitated with Ca_v_α [21]. Ca_v_α_1_ subunits have an oncogenic influence in cancer [15]. Research into Ca_v_ auxiliary subunits in cancer is a growing field, but it appears Ca_v_ auxiliary subunits have both oncogenic and tumour-suppressive effects.

### 2.1 Ca_V_β

The VGCC β-subunits are cytoplasmic proteins that interact with the α_1_ DI-DII intracellular linker region [[22], [23], [24]]. β-subunit binding enhances membrane expression of α_1_ subunits [25,26], however the mechanism by which this occurs has not yet been elucidated. It is thought that β-subunit binding prevents ER retention and the subsequent degradation of Ca_v_2.2, resulting in a higher proportion of Ca_v_2.2 at the plasma membrane [25,27]. However, membrane targeting of the DI-DII linker of Ca_v_2.2 via an inserted palmitoylation motif still results in ER retention and degradation, leading to the hypothesis that Ca_v_β subunits are required for correct folding, and thus membrane insertion, of functional α_1_ subunits [28]. The impact on electrophysiological properties of α_1_ subunits by Ca_v_βs is complex. In general, Ca_v_βs increase current density and regulate activation/inactivation kinetics. For instance, disruption of the Ca_v_β_3_-Ca_V_2.2 interaction by a small molecule inhibitor results in a decrease in current density and a depolarised shift in the voltage threshold of activation and inactivation [29]. In comparison, Ca_v_β_2_ enhances the current density more than Ca_v_β_3_, potentially through increased membrane expression as Ca_v_β_2a_, unlike Ca_v_β_3_, contains a palmitoylation site [30]. Additionally, forced membrane localisation of Ca_v_β_3_ using the N-terminal Lyn sequence enhanced the current density relative to WT- Ca_v_β_3_ [30]. The complexity arises in the differential sensitivity to PIP_2_-mediated modulation of different Ca_v_βs [30,31], competition for α_1_-binding between Ca_v_β subunits [32], the spectrum of functionally-distinct Ca_v_β splice variants [33,34], and the opposing impacts on α_1_-function by the different domains within the Ca_v_β protein [35].

Ca_v_βs are functional independent of direct α_1_ association. All Ca_v_βs demonstrate nucleus localisation, Ca_v_β_4_ particularly within nucleoli, and gene expression regulation [[36], [37], [38], [39]]. All Ca_v_βs also contain a Src homology 3 domain capable of regulating endocytosis via interaction with dynamin and can interact with small GTPases [40,41]. Ca_v_βs show subunit-specific function as well, for instance Ca_v_β_1_ is expressed in muscle progenitor cells (MPCs) earlier than Ca_v_1.1, where it regulates proliferation and directly suppresses myogenin expression. Accordingly, Ca_v_β_1_ knockout mice demonstrate impaired muscle development [36,42]. Similarly, Ca_v_β_2_ is required for ventricle cell proliferation and heart development in zebrafish, although pharmacological VGCC inhibition caused a similar phenotype, suggesting Ca_v_β_2_ may be functioning in an α1-dependent manner [43]. Ca_v_β_2_ is also required for depolarisation-induced c-Fos and meCP2 activation, which intriguingly was shown to be independent of Ca^2+^ influx [37]. Ca_v_β_4_ regulates cell proliferation *in vitro* [44], downregulates Wnt signalling via sequestration of the Wnt pathway effector TCF4 [39], and regulates gene expression via various interacting partners [45,46]. Interestingly, the nuclear localisation of Ca_v_β_4_ was inhibited when co-expressed with Ca_v_1.1 and only upon depolarisation and the presence of extracellular Ca^2+^ did Ca_v_β_4_ interact with its nuclear signalling partner, B56δ [45].

Owing to its role in driving cellular functions such as proliferation and migration, it is perhaps no surprise that Ca_V_α_1_ expression is increased in various cancers [[47], [48], [49]]. However, much research has also been dedicated to evaluating the involvement of Ca_v_ auxiliary subunits in cancer. Ca_v_β_1_ expression is upregulated in colon cancer [50], Ca_v_β_2_ mutations are seen in bladder cancer [51] and increased Ca_v_β_3_ expression is observed in patients with recurrent non-small cell lung tumours compared to recurrence-free patients [52]. Furthermore, expression of Ca_v_β_1_ and Ca_v_β_3_ are included in proposed high-risk gene signatures that correlate with decreased patient survival in colon and recurring non-small cell lung cancer [50,52]. However, the aforementioned studies are largely limited to statistical observations based on tissue sequencing data that identified altered Ca_v_β RNA expression as a high-risk prognostic marker [[50], [51], [52]]. Chen et al. (2016) offered additional pathophysiological justification for increased Ca_v_β_2_ expression in cancer, by observing an enrichment in mutations of genes, including *CACNB2* which encodes Ca_v_β_2_, involved in NCAM-mediated neurite outgrowth [51].

### 2.2 α_2_δ

The Ca_V_ α_2_δ subunit has a unique structure compared to other auxiliary subunits. The translated polypeptide is proteolytically cleaved into two separate proteins, α_2_ and δ, which remain coupled by a disulphide bond [53]. The α_2_ segment is extracellular while the δ-subunit remains associated with the membrane via a GPI-anchor [54]. α_2_δ and Ca_V_β subunits can both induce surface expression of α_1_, but also function synergistically to maximise α_1_ surface expression and Ca^2+^ current [26,55,56]. Preventing proteolytic cleavage of the α_2_δ_1_ proprotein reduces both Ca_v_2.2 surface expression and presynaptic Ca^2+^ influx in hippocampal neurons [57] and site-directed mutagenesis of either cysteine residue involved in the disulphide interaction, which results in a dissociation of α_2_, reduces the whole-cell Ca^2+^ current [53]. Similarly, digestion of the GPI anchor of α_2_δ_3_, by prokaryotic phosphatidylinositol-phospholipase C, results in a release of the α_2_δ from the membrane and a decreased Ca^2+^ current [54]. Both these results suggest an intact α_2_δ subunit is required at the membrane to induce and sustain the α_2_δ-mediated regulation of α_1_ subunits. In addition to its role in trafficking, α_2_δ has been proposed to stabilise α_1_ at the membrane by reducing internalisation and in targeting α_1_ to detergent-resistant membranes [54,58]. Phenotypes of α_2_δ knockout mice have been very informative, both α_2_δ_1_ and α_2_δ_3_ have thus been implicated in neuropathic pain, with α_2_δ_1_-overexpressing mice demonstrating hyperalgesia [59] and α_2_δ_3_ -knockout mice demonstrating an enhanced insensitivity to pain [60]. Mice deficient in α_2_δ_2_, the isoform found overwhelmingly in cerebellar Purkinje neurons, present with seizures and ataxia [61]. Gabapentin, used in the treatment of epilepsy and neuropathic pain, preferentially binds to α_2_δ_1/2_ and lowers α_2_δ surface expression, demonstrating that the α_2_δ auxiliary subunit is a druggable target [[62], [63], [64]]. All α_2_δ subunits are involved in synaptogenesis, but potentially through different mechanisms [65]. α_2_δ_1_ promotes cortical synaptogenesis, independently of Ca^2+^ influx, through binding to secreted astrocytic thrombospondin in the postsynaptic membrane and promoting actin remodelling via Rac-1 [66], whereas loss of α_2_δ_4_ causes impaired retinal synaptogenesis, which correlates with a decrease in presynaptic Ca_v_1.4 [67,68].

More is known about the involvement of α_2_δ subunits in cancer compared to the other Ca_v_ auxiliary subunits. Increased α_2_δ_1_ expression occurs in both ovarian and hepatocellular tumour-initiating cells and correlates with decreased overall survival and a shorter progression-free survival in clinical ovarian samples [[69], [70], [71]]. Zhao et al. developed a monoclonal antibody against α_2_δ_1_, 1B50-1 [71]. Sorting of a 1B50-1-positive subpopulation of Hep-11 cells, a hepatocellular carcinoma (HCC) cell line, resulted in a subset of cells that initiated tumour formation in all implanted mice, whereas the 1B50-1-negative subpopulation failed to form any tumours. Furthermore, 62/86 of HCC samples were 1B50-1-positive compared to 0/6 normal tissue samples. *in vivo* experimentation demonstrated that administering 1B50-1 reduced tumour volume following implantation of two HCC cell lines and increased survival, especially when co-administered with doxorubicin, compared to doxorubicin or 1B50-1 alone. Lastly, *in vitro* work in the same study demonstrated α_2_δ_1_ to be involved in maintaining cell viability and spheroid formation, via increasing Ca^2+^ influx through L-type and N-type Ca^2+^ channels and MAPK signalling [71]. In non-small cell lung cancer cells, α_2_δ_1_ expression confers radioresistance *in vitro*, by enhancing the DNA repair response, and chemoresistance *in vivo*, potentially through MAPK signalling [72,73]. In addition, various miRNAs that are downregulated in cancer target α_2_δ_1_ expression, including hsa-miR-208a-3p and hsa-miR-1207-5p in medulloblastoma [74], and miR-107 in chronic myeloid leukaemia (CML) [75]. Overexpressing miR-107 promotes differentiation in CML cell lines, which is reversed when expression of α_2_δ_1_ is restored [75].

The involvement of α_2_δ_2_ in cancer is complex, as α_2_δ_2_ can be both oncogenic and tumour suppressive [76,77]. α_2_δ_2_ was initially identified as a potential tumour suppressor gene as it is encoded by *CACNA2D2*, which is absent in the 3p21.3 chromosomal deletion commonly observed in lung and breast cancer [78]. Similarly, *CACNA2D2* is deleted in cervical carcinoma [79], is commonly methylated in head and neck squamous cell carcinoma [80], is downregulated in lung squamous cell carcinoma via miR-205 [81], and its expression correlates with improved survival in patients with lung adenocarcinoma [82]. Functionally, *in vitro* experiments using various non-small cell lung cancer cell lines have demonstrated that overexpression of α_2_δ_2_ induces apoptosis via mitochondrial cytochrome-c release and subsequent caspase activation [77]. In contrast, α_2_δ_2_ overexpression occurs in prostate tumours [76] and in insulin-secreting pancreatic adenomas, where elevated intracellular Ca^2+^ is known to stimulate β-cell proliferation [83]. Furthermore, α_2_δ_2_ overexpression in prostate cancer cells induces tumourigenesis and angiogenesis in mice, which is treatable by administering the α_2_δ_2_ inhibitor, gabapentin [76].

Conversely, α_2_δ_3_ is considered a tumour suppressor gene, as downregulation or deletion is seen in nasopharyngeal cancer [84], breast cancer [85], oesophageal squamous cell carcinoma [86,87], gastric cancer [88,89], lung cancer [90] and cholangiocarcinoma [91]. Mice implanted with cancer cells overexpressing α_2_δ_3_ show a decreased tumour volume, compared to implanted control cells, in nasopharyngeal cancer [84], oesophageal cancer [87] and glioma [92] models. The consensus mechanism points towards an inhibition of motility and invasion by α_2_δ_3_, and induction of apoptosis through an increase in intracellular Ca^2+^, leading to mitochondria-induced apoptosis [84,87,92].

### 2.3 Ca_V_γ

The interaction between Ca_V_γ-subunits and α_1_ subunits is less well understood. Ca_v_γ-subunits were originally identified following immunoprecipitation of the skeletal muscle 1,4-dihydropyridine (DHP) receptor (later known as L-type VGCCs), which yielded γ_1_ as a binding partner [93,94]. Following the discovery of Ca_V_γ_1_, seven more Ca_v_γ-subunits were identified by homology studies [[95], [96], [97], [98]]. Ca_v_γ_2_ and Ca_v_γ_3_ have been shown to associate with Ca_v_2.1 [99], Ca_v_γ_2-4_ to Ca_v_2.2 [99] and Ca_v_γ_6_ to Ca_v_3.1 [100]. Using cryo-electron microscopy, the γ-subunit was predicted to interact with the Ca_v_1.1 voltage-sensing domain (S4) of domain IV [24]. However, the α_1_-γ coupling remains contentious as more recent efforts failed to precipitate a Ca_v_γ-subunit with Ca_v_2. Further, Ca_v_γ_2_ can regulate Ca_v_2.2 indirectly, suggesting a direct coupling may not be necessary for Ca_v_γ-induced channel modulation [21,101]. Ca_v_γ-subunit mRNA is expressed in skeletal muscle (γ_1,6,7_) and brain (γ_2-8_) as well as other tissues such as kidney, liver, colon, testis and lung [98]. Functionally, Ca_v_γ-subunits negatively regulate VGCC-mediated Ca^2+^ influx by decreasing channel expression and current amplitude [102], hyperpolarising the voltage threshold of inactivation, accelerating channel inactivation [103], and increasing the time taken for recovery from inactivation [96]. Ca_v_γ-induced regulation of Ca^2+^ influx observed at the cellular level is supported by the *Stargazer* mouse mutant, which lacks Ca_v_γ_2_ and presents with ataxia and absence seizures [104]. Interestingly, a subclass of Ca_v_γ-subunits, γ_2/3/4/5/8_ (known as transmembrane AMPA receptor regulatory proteins [TARPs]), which localise to the brain [105], interact with ionotropic AMPA receptors and induce membrane localisation [106,107]. Other functions of γ-subunits include Ca_v_γ_7_-induced neurite outgrowth in superior cervical ganglion neurons [108] and Ca_v_γ_2_-induced synaptogenesis [109].

Aberrant Ca_v_γ expression is seen in various cancers, including increased Ca_v_γ_1_ in early progressing human epidermal growth factor-positive (HER2+) metastatic breast cancer [110], increased Ca_v_γ_4_ in bladder squamous cell carcinoma [111] and increased Ca_v_γ_7_ in leiomyoma via downregulation of miR-197 [112]. Furthermore, a prediction algorithm using a dataset of 1.7 million cancer mutations identified Ca_v_γ_3_ as a putative oncogene [113]. Similar to Ca_v_β, the functional role of Ca_v_γ in cancer is not yet clear. However, a Ca_v_γ_4_ mutation appears in a cluster of mutations involved in MAPK signalling [111], suggesting a possible role in regulation of mitogenesis.

In summary, although Ca_v_α_1_ subunits have an oncogenic role [15], it is not yet clear whether Ca_v_ auxiliary subunits function through Ca_v_α_1_ or have secondary functions in cancer, or both. Given that Ca_v_β and Ca_v_γ are both oncogenic but have antagonistic effects on α_1_ function, and Ca_v_α_2_δ can be oncogenic or tumour suppressive, it would seem that the involvement of auxiliary subunit-mediated Ca^2+^ influx in cancer is tumour type/stage-specific, dependent on the expression profile of other subunits, or subordinate to a secondary function of the auxiliary subunit. Ca_v_ auxiliary subunits have functions, potentially α_1_-independent, that could contribute to oncogenesis and tumour progression. All Ca_v_βs regulate gene expression and interact with small GTPases [[36], [37], [38],^40^,^41^,^44^]. Ca_v_β_1_ and Ca_v_β_2_ are also essential for maintaining proliferation and cellular plasticity during development [36,43]. The TARP family of Ca_v_γs induce AMPA receptor membrane trafficking [107], a receptor with an emerging involvement in cancer [114,115], and Ca_v_γ_4_ and Ca_v_γ_7_ induce transcellular adhesion and neurite outgrowth respectively [108,109]. α_2_δ_1_ is also involved in transcellular adhesion [66]. Furthermore, increased Ca^2+^ conductance potentially underpins both the oncogenic function of α_2_δ_1_ and α_2_δ_2_ [71,83] and the tumour suppressive function of α_2_δ_2_ and α_2_δ_3_ [77,92].

## 3 K^+^ channels

**Fig. 2:**
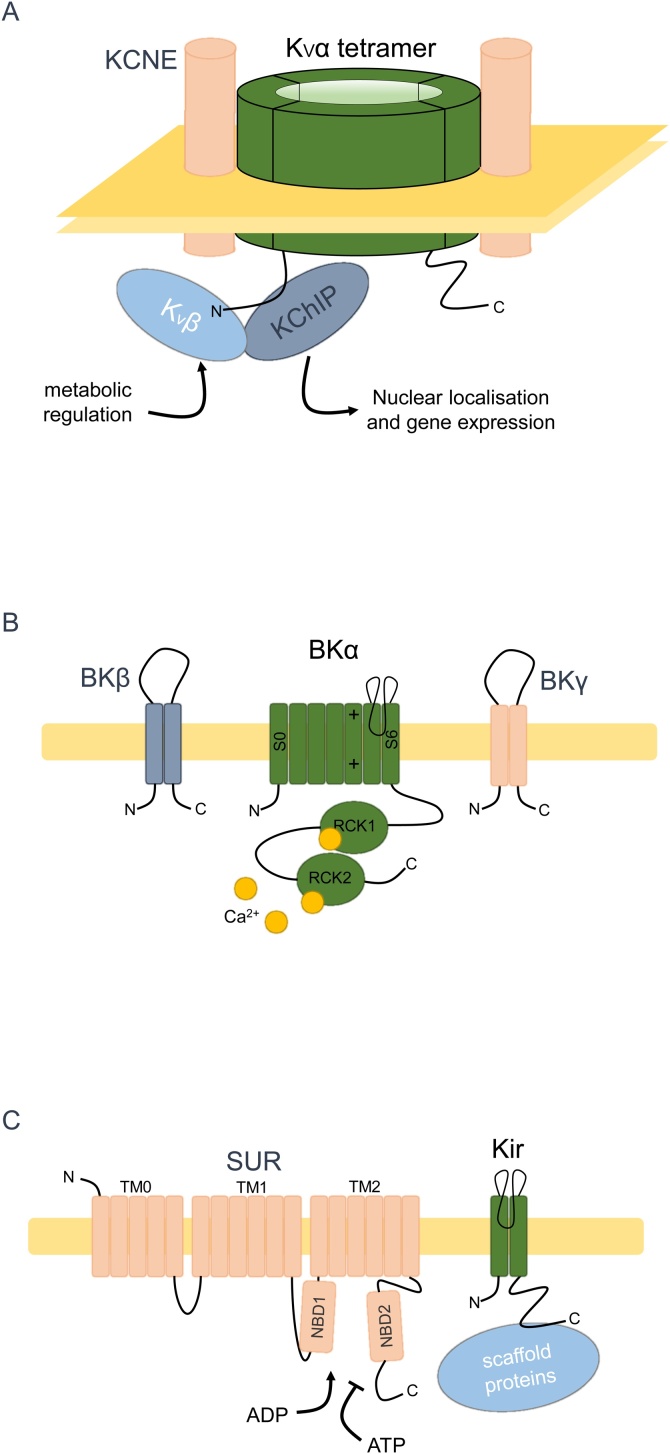
K^+^ channel auxiliary subunits. (**A**) Voltage-gated K^+^ channels (VGKCs). The conducting subunit, K_v_α forms tetramers within the membrane that are accompanied and functionally modulated by four K_v_βs (for K_v_1), four KChIPs (for K_v_4) or two KCNEs (K_v_7.1) [[119], [120], [121], [122]]. The function of K_v_β is modulated by pyridine nucleotides [143]. KChIPs are involved in regulating gene expression [173]. (**B**) Large conductance Ca^2+^-activated K^+^ (BK) channels. BK channels consist of a K^+^-conducting, seven-pass (S0-S6) membrane protein subunit (BKα/Slo) accompanied and modulated by dual-pass BKβ and BKγ [182,187]. S0 of BKα is required for interaction with BKβ, S4 is involved in voltage-sensing, the pore region is formed by the linker of S5-6 and an enlarged C-terminus containing two RCK (regulator of conductance of K^+^) domains sense intracellular Ca^2+^ [330]. (**C**) Inwardly rectifying K^+^ (K_ir_) channels. Tetrameric K_ir_6 subunits, containing the K^+^-conducting pore, are functionally regulated at the membrane by 17-pass SUR subunits (1:1 stoichiometry), which confer ATP-sensitivity onto K_ir_6 via NBDs (nucleotide binding domains) [196]. K_ir_1-4 can be bound and modulated by various C-terminal binding proteins [331].

K^+^ channels represent an extensive superfamily of channels, many of which have been implicated in regulating key elements of tumour progression [[116], [117], [118]]. Here, we focus on the function and involvement in cancer of the auxiliary subunits of the voltage-gated K^+^ channel (VGKC), BK channel and K_ir_ channel complexes (Fig. 2A-C). VGKC α-subunits represent a diverse family of forty K^+^-conducting proteins, K_v_1-12.*x*, which conduct an outward K^+^ current in response to depolarisation of the membrane potential. Three classes of VGKC auxiliary subunits have been identified: K_v_β_1-3_, KChIP1-4, and KCNE1-5 which canonically interact with K_v_1, K_V_4, and K_v_7.1 respectively [[119], [120], [121], [122]], although K_v_βs and KCNEs interact with other VGKC α-subunits and K_V_βs also interact with TRPV1 and K_2_P2.1 [[123], [124], [125], [126]]. The activity of K_v_1 [116,127], K_v_4 [128], and K_v_7.1 [129] is upregulated in various cancers. However, the expression pattern of VGKC auxiliary subunits in cancer is more complex.

### 3.1 K_v_β

K_v_β subunits are cytoplasmic proteins, which form homo- or heterotetramers [130] that are involved in trafficking of K_v_1 and K_v_4.3 to the cell surface [[131], [132], [133]]. Additionally, K_v_β_2_ is involved in targeted axonal trafficking of K_v_1.2 and K_v_β_1_ differentially regulates the K_v_ composition in ventricular myocytes [134,135]. K_v_β_1_ and K_v_β_3_ modulate VGKC α-subunits via an N-terminal ball domain, which permits rapid inactivation of delayed-rectifying K_v_1 α-subunits [136,137]. K_v_β_1_ also slows deactivation, accelerates slow inactivation and hyperpolarises activation of K_v_1.2 [138]. K_v_β_2_ lacks the ability to inactivate delayed-rectifying K_v_1 channels, but does hyperpolarise channel activation [139]. K_v_β_1_ and K_v_β_2_ are both expressed in developing rat heart and skeletal muscle and during induced myogenesis of L6E9 cells [140]. Furthermore, deletion of K_v_β_1_ results in aberrant cardiac electrical activity and cardiac hypertrophy in female mice [141]. K_v_β_2_ deletion leads to reduced K_v_1.5 surface expression in coronary arterial myocytes and a reduction in total skeletal muscle volume, potentially mediated through downregulation of Pax7 and upregulation of NEDD4 [133,142]. Interestingly, K_v_βs are part of the aldo-keto reductase (AKR) superfamily owing to their C-terminal AKR domain. The AKR domain allows for binding and functional modulation by pyridine nucleotides (NAD and NADP). NADP^+^ inhibits K_V_β_1_- and K_V_β_3_-mediated inactivation of K_v_1.5 as well as inhibiting K_v_β_2_-mediated hyperpolarisation of K_v_1.5 activation [143,144].

Evidence suggests that K_v_βs are downregulated in cancer. K_v_β_1_ is downregulated in malignant thyroid carcinomas relative to benign thyroid adenomas [145,146]. The gene encoding K_v_β_2_ is the most significant site of methylation in non-functional (non-hormone secreting) pituitary adenoma compared to functional (hormone-secreting) adenomas and is one of the genes ablated in the common 1p36.3 chromosome deletion seen in neuroblastoma [147,148]. Methylation of the promoter of the gene encoding K_v_β_3_ is seen in oral squamous cell cancers relative to adjacent normal tissue [149]. Together, these data suggest K_v_βs are tumour suppressor genes, but in depth *in vitro* and *in vivo* characterisation of K_v_β in cancer is still currently lacking.

### 3.2 KCNE

KCNEs are single-pass transmembrane proteins that interact primarily with K_v_7; two KCNEs interact with tetrameric K_v_7 [150]. *In vitro* studies document a range of effects of KCNEs on K_v_7.1. For example, KCNE1 and KCNE3 both increase surface expression and current density, while KCNE4 and KCNE5 have no effect on current density [151]. KCNE2 and KCNE3 interaction with K_v_7.1 produces voltage-insensitive channels and all KCNEs depolarise the activation voltage of K_v_7, with KCNE4 and KCNE5 depolarising activation to a non-physiological membrane potential [151]. K_V_7.1 has a well-established role in cardiac rhythm and in regulating osmotic and salt transport across gastrointestinal, cochlear and renal epithelia; this is reflected in *Kcne1* knockout mice demonstrating atypical QT intervals, hair cell degeneration, impaired renal fluid, glucose and electrolyte uptake, and faecal Na^+^ and K^+^ wasting [[152], [153], [154], [155]]. Furthermore, mutations in *KCNE1* underlie Long QT Syndrome 5 and Jervis and Lange-Nielsen syndrome, a disorder characterised by deafness and cardiac arrhythmia [156,157].

With regard to cancer, KCNE1-3 are expressed in uterine cancer cell lines, in which they influence proliferation [158] and a 5-fold and 3-fold upregulation of KCNE3 and KCNE4 respectively has been reported in gliobastoma datasets [159]. Paradoxical to the upregulation of KCNE1 in uterine cancer cell lines, KCNE1 overexpression in an astroglioma cell line (U87-MG) induces apoptosis and *KCNE1* is one of the four genes deleted in the 21q22.12 microdeletion which causes a predisposition to acute myelogenous leukaemia [160,161]. The apoptotic influence of KCNE1 in U87-MG cells is proposed to occur through canonical K^+^ efflux through K_v_7.1, inducing decreased cytoplasmic K^+^, a known apoptotic trigger [160,162], whereas KCNE1 induces uterine cancer cell proliferation via modulation of HERG channels [158,163]. HERG channels induce proliferation in a range of cell lines and HERG channel inhibition decreases MAPK phosphorylation and c-fos expression in MDA-MB-435S cells [164]. Out of all the K_v_ auxiliary subunits however, KCNE2 has the most established link to cancer. KCNE2 downregulation is observed in gastric cancer tissue and gastric cancer cell lines, correlates with gastritis cystica profunda development (preneoplastic condition characterised by large gastric cysts) and is a risk factor in gastric cancer stratification [[165], [166], [167]]. Furthermore, *Kcne2* knockout mice display a 6-fold increase in stomach size, an upregulation of Ki67 and Cyclin D1 in gastric mucosa, an increase in the metaplastic marker TFF2, pyloric adenomas and neoplastic invasion compared to wild-type mice [168]. Overexpression of KCNE2 in the SGC7901 gastric cancer cell line reduces proliferation and significantly reduces xenograft tumour volume compared to parental SGC7901 cells [167].

KCNE2-K_v_7.1 complexes, in the apical membrane of non-excitable gastric parietal cells, are essential for maintaining acidification of the stomach, as KCNE2 transforms K_v_7.1 to a constitutively open channel that is potentiated by extracellular H^+^ [169]. Luminal K^+^ released by KCNE2-K_v_7.1 is then recycled back into the parietal cell, in exchange for H^+^, via the H^+^/K^+^ ATPase, resulting in gastric acidification [169,170]. *Kcne1* knockout mice demonstrate reduced H^+^ secretion, reduced gastric acidification, gastric hyperplasia and atypical K_v_7.1 localisation [170]. However, it is not yet known whether KCNE2 downregulation contributes to gastric cancer progression through a failure to acidify the lumen of the stomach or via its role in regulating tumour cell proliferation.

### 3.3 KChIP

Ca^2+^-sensing K_v_ channel interacting proteins (KChIPs) are involved in K_V_4 channel modulation. KChIPs increase surface channel density, hyperpolarise the voltage of activation, slow inactivation and accelerate the recovery from inactivation [119,171]. KChIPs were identified by a yeast 2-hybrid screen searching for interaction partners with K_v_4.2/3 N-termini [119]. Interestingly, KChIP3 was already known as calsenilin/downstream regulatory element antagonistic modulator (DREAM). KChIP3/DREAM plays a key role in differentiation and apoptosis independently of K^+^ channels [172]. DREAM binds upstream genetic elements (DRE sites) as a tetramer and represses transcription of the downstream gene until upon Ca^2+^ stimulation, DREAM tetramers dissociate from DNA allowing gene transcription [173]. Despite KChIP3 being the first Ca^2+^-sensing transcriptional repressor identified, the other KChIPs are also capable of DRE-site binding [174]. DREAM expression is required for maintenance of human embryonic stem cell pluripotency; DREAM knockdown by siRNA results in an increase in apoptosis and spontaneous differentiation [172]. Potentially independent of its nuclear role, DREAM expression induces Ca^2+^-mediated apoptosis possibly through sequestration of hexokinase I from mitochondria [175,176]. Additionally, DREAM expression induces process outgrowth in pheochromocytoma PC12 cells by RhoA inactivation and induces thrombus formation in anucleate platelets via PI3K stimulation [177,178]. There is currently limited evidence of a role for KChIPs in cancer. However, one study identified KChIP4 gene disruption in a renal cancer cell chromosomal break [179]. In addition, KChIP1 upregulation and KChIP3 downregulation have been shown in glioblastoma multiforme, with KChIP2 upregulation correlating with decreased survival for glioblastoma patients [180]. The involvement of KChIP3/DREAM in regulating differentiation, apoptosis, transcellular adhesion and process outgrowth suggests cancer-expressed or downregulated KChIPs could be a worthwhile subject of further study.

### 3.4 BK channels

Large conductance Ca^2+^-activated K^+^ (BK) channels are seven membrane-pass K^+^ channels that conduct a particularly large outward K^+^ current synergistically in response to membrane depolarisation and a rise in intracellular Ca^2+^ ([Ca^2+^]_i_) [181]. BK channels can be stimulated by depolarisation or increased [Ca^2+^]_i_ alone, however the required membrane potential (V_1/2_ = 168 mV at [Ca^2+^]_i_ = 0) or [Ca^2+^]_i_ (EC_50_ ≥10 μM at resting membrane potential) are out of physiological range [182]. BK channels are expressed in most tissues and are involved in a range of functions, such as learning and memory [183], pain modulation [184] and blood pressure regulation [185]. BK channels are upregulated in glioblastoma primary cells and promote proliferation and invasion [117,186]. BK channel function is modulated by two groups of auxiliary subunits- BKβ_1-4_ and BKγ_1-4_, both double-pass membrane proteins. BKβ_1_ and BKβ_2_ increase Ca^2+^ sensitivity [187], BKβ_2_ hyperpolarises and accelerates channel activation [188], BKβ_3_ depolarises channel activation [188] and BKβ_4_ hyperpolarises channel activation whilst simultaneously inhibiting channel opening at low [Ca^2+^]_i_ but enhancing activation at high [Ca^2+^]_i_ [189]. BKγ subunits hyperpolarise BK channel activation [190]. BKγ_1_ hyperpolarises channel activation to such an extent (−140 mV in LNCaP prostate cancer cells) that BK channels open without the need for increased [Ca^2+^]_i_ at resting membrane potentials [182].

Despite the extensive involvement of BK channels in a range of physiological processes, the link between BK channel auxiliary subunits and cancer is still very tentative, with thus far only BKγ_1_ implicated. There are conflicting reports on the involvement of BKγ_1_ (also known as LRRC26 and CAPC) in cancer. BKγ_1_ is upregulated in the MDA-MB-456 breast cancer cell line and in metastatic secondary breast cancer tumours compared to the primary tumour of a single patient [191]. BKγ_1_ is also upregulated in many breast and prostate cancer cell lines and breast, prostate, colon and pancreatic samples [192,193]. However, BKγ_1_ is frequently methylated in triple-negative breast cancer specimens and cell lines and siRNA knockdown of BKγ_1_ in the triple-negative HCC70 breast cancer cell line enhances anchorage-independent growth, invasion, migration, and NF-κB activity [194]. Similarly, knockdown of BKγ_1_ expression enhances anchorage-independent growth in LNCaP cells and overexpression of BKγ_1_ in the triple-negative MDA-MB-231 breast cancer cell line downregulates NF-κB activity and inhibits tumourigenesis and metastasis in nude mice [195]. Furthermore, BKγ_1_ expression is lowest in poorly differentiated and highly invasive prostate and breast cancer lines [195]. Thus, BKγ_1_ appears to have oncogenic and tumour-suppressive function depending on the cancer type. At this stage, the mechanism by which BKγ_1_ performs these functions in cancer cells is unclear. BK channels may thus perform multiple functions in cancer cells, dependent on, or independent of, BKγ_1_.

### 3.5 K_ir_ channels

Inwardly-rectifying K^+^ (K_ir_) channels are double pass membrane proteins which form tetramers in the membrane [196]. K_ir_ channels lack a voltage sensor domain. I_Kir_ is instead dictated by the electrochemical gradient and an increasing intracellular blocking of the pore when the membrane potential (E_m_) > E_K_, resulting in an inward I_K_ when E_m_ < E_K_ and an outward I_K_ when E_m_ > E_k_, which is progressively blocked as E_m_ rises [197]. K_ir_ channels are therefore important for maintenance of the hyperpolarised resting membrane potential and regulating activity in excitable cells, such as vascular smooth muscle [198], central neurons [199] and cardiomyocytes [200]. Subfamilies of K_ir_ channels exist that are ATP-sensitive (K_ATP_ channels; K_ir_6.*x*) and G-protein gated (G-protein inwardly rectifying K^+^ channels- GIRKs; K_ir_3.*x*) [201,202]. K_ATP_ channels are inhibited by ATP/stimulated by ADP. They function as metabolic sensors, for instance in smooth muscle where K_ATP_ channels regulate vascular tone [203]. GIRKs facilitate G-protein-mediated inhibitory neurotransmitter signalling, such as GABA signalling [204,205].

Certain K_ir_ channels are regulated by auxiliary subunits. K_ir_6 binds sufonylurea receptors (SUR) 1 or 2 in an octameric conformation (tetrameric K_ir_6 plus tetrameric SUR) to form a K_ATP_ channel [196]. Channel assembly is required before K_ATP_ is released from the endoplasmic reticulum [206]. SUR subunits impart differential sensitivity to ADP/ATP and are the binding target of sulfonylureas, a common form of treatment for type 2 diabetes mellitus [207,208]. SUR1 is overexpressed in cerebral metastases where it decreases vascular permeability [209]. Resveratrol binds to and inhibits SUR1, inducing apoptosis in HEK293 cells, suggesting a potential pro-survival function of SUR1 [210]. SUR2B expression is present in leiomyoma and metastatic breast cancer cells and glibenclamide, a sulfonylurea targeting SUR proteins, inhibits proliferation in these cells [211,212]. SUR2 expression, along with K_ir_6.2, is upregulated in cervical cancer biopsies [213]. In addition, the effectiveness of glibenclamide at inhibiting proliferation correlates with the K_ir_6.2 expression of the cell line tested, suggesting proliferation is dependent on SUR and K_ir_6.2 activity [213]. Glibenclamide also inhibits proliferation in MDA-MB-231 breast cancer cells, inducing G0/G1 cell cycle arrest through an upregulation of P27 and reduction of cyclin E [212]. Treatment of MDA-MD-231 cells with the K_ATP_ channel opener, minoxidil, conversely induces proliferation, suggesting K^+^ influx underlies K_ATP_-regulated proliferation [212]. Glibenclamide treatment also prevents tumour growth *in vivo* in Sprague-Dawley rats treated with N-nitroso*-N-*methylurea [214]. Furthermore, in insulinoma, a pancreatic β-cell cancer characterised by insulin release, which is regulated by K_ATP_ channels, SUR1 expression is increased [215]. In summary, SUR subunits appear to play an oncogenic role in a K_ir_-dependent manner.

## 4 Na^+^ channels

**Fig. 3:**
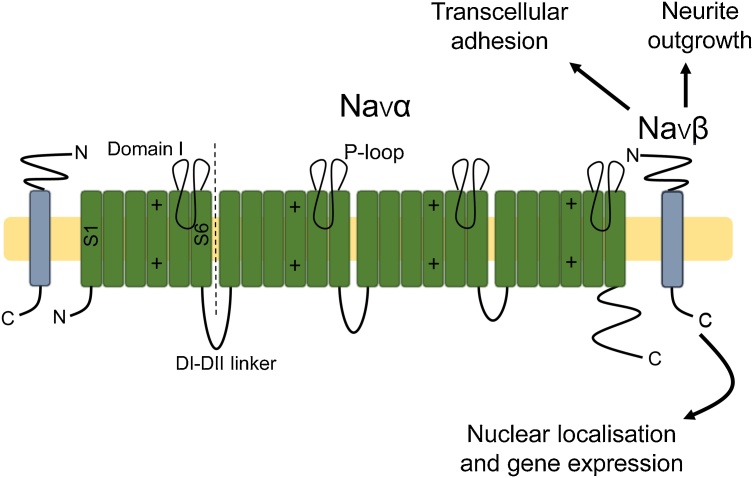
Voltage-gated Na^+^ channel auxiliary subunits. Voltage-gated Na^+^ channels (VGSCs) contain a conducting Na_v_α subunit and auxiliary Na_v_β subunits. Na_v_α consists of four domains (domains I-IV), each containing six segments (S1-S6). The voltage-sensing domain is found within S4 of each domain and the pore consists of the P-loop found between S5-6 of each domain. Na_v_βs function as cell adhesion molecules via an extracellular immunoglobulin domain [238,239,332]. Na_v_βs also induce neurite outgrowth and migration [245] and the intracellular domain of Na_v_β_2_ has putative transcription regulation function [248].

There is a growing body of evidence supporting a role for Na^+^ channels in regulating various aspects of cancer progression [216,217]. With regard to auxiliary subunits, however, only those of the VGSC have been characterised to date and will therefore be the focus of this section (Fig. 3).

### 4.1 Voltage-gated Na^+^ channels

VGSCs conduct an inward Na^+^ current in response to membrane depolarisation [218]. VGSCs are composed of a pore-forming α-subunit (Na_v_1.1–1.9) and auxiliary β-subunits (Na_v_β_1_-Na_v_β_4_). Na_v_βs are single pass transmembrane glycoproteins that bind Na_v_α covalently, in the case of Na_v_β_2_ and Na_v_β_4_ [219,220], or non-covalently, in the case of Na_v_β_1_ and Na_v_β_3_ [[221], [222], [223]]. I_Na_ is responsible for propagation of action potentials and mutations in Na_v_βs underlie certain types of epilepsy [224] and cardiac arrhythmia [225]. Na_v_β_1-3_ traffic Na_v_α to the cell surface [[226], [227], [228]] and all Na_v_βs increase I_Na_ [[229], [230], [231]]. Na_v_βs induce other changes in Na_v_α gating kinetics, including accelerated recovery from inactivation [232,233] and accelerated inactivation [230,234]. Na_v_βs can both positively and negatively shift the voltage of activation [235,236] and inactivation [222,226], possibly dependent on endogenous expression of Na_v_ subunits and other Na_v_-interacting proteins in the experimental system used. Na_v_βs are also cell adhesion molecules, owing to the presence of an extracellular immunoglobulin loop [[237], [238], [239], [240]], which permits Na_V_β-mediated neurite outgrowth [[241], [242], [243], [244]]. Na_V_β1 plays an important role in regulating neuronal migration in CNS development, particularly in the cerebellum [14,245], and Na_V_β_2_ promotes dendritic expansion during hippocampal development via a Na_v_α-independent mechanism [243]. Na_V_β subunits are also substrates for proteolytic processing by secretases [246,247] and evidence suggests that the cleaved intracellular domain of Na_V_β_2_ shuttles to the nucleus to regulate expression of α-subunit genes [248].

Emerging evidence suggests that Na_v_βs play diverse functional roles in cancer. Na_v_β_1_ is upregulated in breast cancer samples and is more highly expressed in strongly metastatic, compared to weakly metastatic, prostate cancer cell lines [249,250]. Overexpression of Na_v_β_1_ in the MDA-MB-231 breast cancer cell line promotes primary tumour growth and metastasis to multiple organs when grafted into mice, compared to parental MDA-MB-231 cells [249]. The Na_v_β_1_-induced increase in primary and secondary tumour growth was accompanied by a decrease in apoptotic cleaved caspase-3 staining, no change in proliferative Ki67 staining, and an increase in endothelial CD31 staining, suggesting increased apoptotic resistance and vascularisation underlie the oncogenic influence of Na_v_β_1_ [249]. *In vitro*, MDA-MB-231-Na_v_β_1_ cells demonstrate increased cell-cell adhesion, VGSC-mediated Na^+^ current and neurite-like process outgrowth, which is reversible by inhibiting I_Na_ [249,251]. Interestingly, MDA-MB-231-Na_v_β_1_ cells show decreased *in vitro* motility and proliferation compared to MDA-MB-231 cells and knockdown of endogenous Na_v_β_1_ in the MCF-7 breast cancer cell line increases cell migration [251]. Similarly, Na_v_β_1_ is also expressed in cervical cancer cells where it inhibits motility [252]. Furthermore, treatment of mouse melanoma B16F10 cells with the anti-cancer polymethoxyflavone, casticin, inhibits cell migration and invasion and causes a concomitant genomic upregulation of *SCN1B* (encoding for Na_v_β_1_) [253]. Na_v_β_1_ therefore appears to have a negative influence on cell behaviour *in vitro* and potentially induces tumour growth and metastasis through an increase in apoptotic resistance and transcellular adhesion.

Na_v_β_2_ also appears to be oncogenic. Na_v_β_2_ expression is increased in strongly metastatic prostate cancer cell lines relative to weakly metastatic cell lines [254]. Perineural invasion is common in invasive prostate cancer, and LNCaP prostate cancer cells overexpressing Na_v_β_2_ demonstrate an increased association with *ex vivo* murine spinal cord axons and an increase in migration, invasion and growth [254,255]. Despite the invasion-promoting behaviour of Na_v_β_2_ *in vitro*, overexpression of Na_v_β_2_ in LNCaP cells inhibits tumour growth, compared to LNCaP cells, when implanted into mice, suggesting the functional contribution of Na_v_β_2_ might be site or stage-specific during cancer progression [255].

Unlike Na_v_β_1_ and Na_v_β_2_, Na_v_β_3_ and Na_v_β_4_ are considered tumour-suppressive. *SCN3B* (encoding for Na_v_β_3_) expression is strongly upregulated by p53 following DNA damage and Na_v_β_3_ expression induces apoptosis and suppresses colony formation in osteosarcoma and glioblastoma cell lines [256]. Na_v_β_4_ expression is downregulated in thyroid and high-grade breast cancer and is associated with favourable survival [231,257]. Downregulation of Na_v_β_4_ in MDA-MB-231 breast cancer cells with shRNA increases primary tumour growth and metastasis in xenograft mice models, relative to MDA-MB-231 cells overexpressing Na_v_β_4_ [231]. Furthermore, loss of Na_v_β_4_ increases Na_v_α-independent RhoA-mediated cancer cell migration and invasion [231]. Na_v_β_4_ also suppresses invasion in cervical cancer cells [252]. Na_v_βs are structurally very similar and generally have a broadly comparable effect increasing I_Na_, so it is intriguing that Na_v_β_1_ and Na_v_β_2_ are oncogenic, whereas Na_v_β_3_ and Na_v_β_4_ are tumour-suppressive. Additionally, both Na_v_β_1_ and Na_v_β_4_ were investigated using the same breast cancer cell, MDA-MB-231, so the endogenous VGSC subunit expression accompanying the Na_v_β-subunit is comparable [231,249]. Both Na_v_β_1_ and Na_v_β_4_ inhibit cell migration *in vitro* and induce neurite outgrowth in developing neurons, thus it is unclear where the functional discrepancy between the two proteins lies [231,241,251,258].

## 5 Cl^−^ channels

**Fig. 4:**
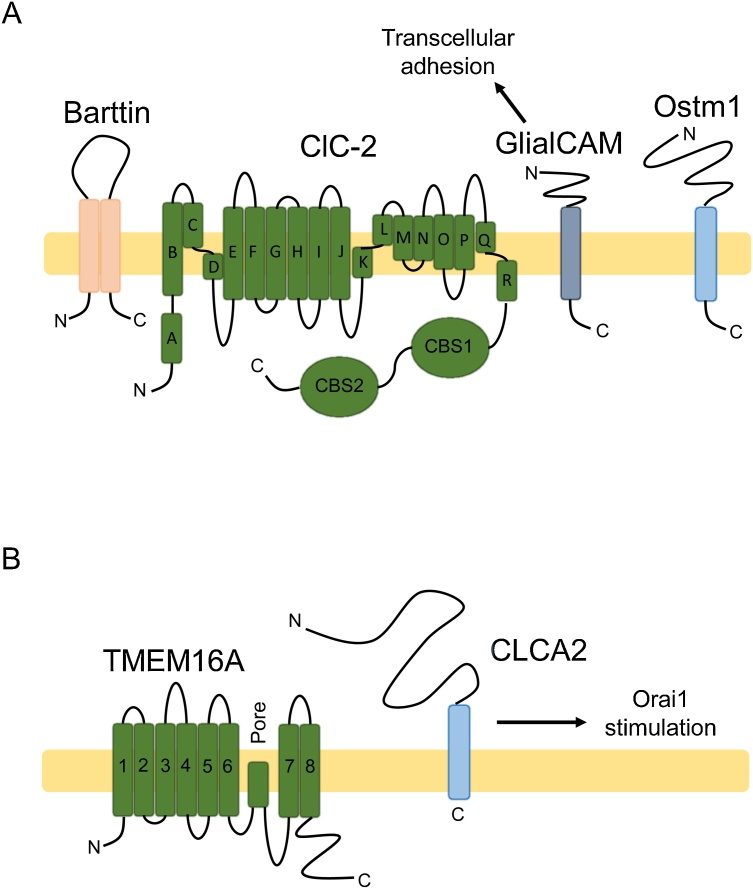
Cl^−^ channel auxiliary subunits. (**A**) CLCs are a subfamily of voltage-sensitive Cl^−^ channels and transporters found at the plasma membrane and internal membranes [13]. Barttin modulates ClC-K, GlialCAM modulates ClC-2 and Ostm1 modulates the intracellular ClC-7 transporter [264,266,267]. CLCs are composed of eighteen helical domains and two C-terminal cystathionine-β-synthase (CBS) domains which facilitate dimerization [333]. Depicted is the plasma membrane ClC-2 which interacts with single-pass GlialCAM, the only ClC auxiliary subunit implicated in cancer [264]. GlialCAM can also function as a cell adhesion molecule [268]. (**B**) Two separate CaCC conducting subunits exist- TMEM16 and Bestrophin. Depicted is eight-pass TMEM16 A which is modulated directly by secreted CLCA1 and indirectly by single-pass CLCA2 [303,305]. CLCA2 stimulates Ca^2+^ store replenishment by interacting with Orai1 and STIM1 [305].

Cl^−^ channels are a family of relatively poorly understood proteins that facilitate transmembrane Cl^−^ transport. Cl^−^ concentration is highest intracellularly and E_Cl_ ˜-30 to −60 mV, so channels conduct an outward Cl^−^ current at resting membrane potentials that can reverse on depolarisation, although inwardly and outwardly rectifying Cl^−^ channels have been identified [13]. Cl^−^ channels are involved in regulating a range of bodily functions, including renal salt retention [259], synaptic inhibition [260], skeletal muscle contraction [261], smooth muscle tone [262] and sperm motility [263]. Various subfamilies of Cl^−^ exist, but only the voltage-gated Cl^−^ channel (CLC) and Ca^2+^-sensitive Cl^−^ channel (CaCC) subfamilies possess auxiliary subunits with a robust link to cancer (Fig. 4A, B).

### 5.1 Voltage-gated Cl^−^ channels

CLCs represent a range of cell surface Cl^−^ channels (ClC-1,2,K) and intracellular Cl^−^ exchangers (ClC-3-7). Some CLCs are regulated by auxiliary subunits; ClC-2 by GlialCAM [264,265], ClC-7 by Ostm1 [266], and ClC-K by Barttin [267]. GlialCAM targets ClC-2 to cell-cell junctions, increases Cl^−^ current (I_Cl_), accelerates I_Cl_ activation, and abolishes ClC-2 inward rectification and pH sensitivity [264]. GlialCAM also functions as a cell adhesion molecule via an extracellular immunoglobulin domain [268,269]. ClC-7 is an intracellular, electrogenic H^+^/Cl^−^ exchanger involved in lysosomal acidification [270]. Interestingly, ClC-7 regulates the trafficking and expression of its auxiliary subunit, Ostm1 [266,271]. Nevertheless, Ostm1 is required to activate ClC-7 function [270]. Barttin traffics ClC-K to the cell surface, resulting in increased I_Cl,_ and abolishes the voltage-dependence of ClC-K [[272], [273], [274]]. Mutations in the gene encoding Barttin are the cause of Bartter syndrome type IV, characterised by hypokalaemia, blood alkalosis and hypotension [275,276]. Knockin mice with the disease-causing Barttin mutation R8L present with reduced plasma membrane Barttin-ClC-K complexes and transepithelial Cl^−^ transport is impaired in the loop of Henle [277].

GlialCAM (also called HepaCAM) was identified as a putative tumour suppressor gene that is silenced in hepatocellular carcinoma [278]. GlialCAM downregulation is observed in liver, bladder, prostate, kidney, breast, uterus, colon, stomach, and rectal cancer biopsies [269,[278], [279], [280], [281], [282]]. Functionally, when GlialCAM is expressed in the liver carcinoma cell line HepG2, cell motility and adhesion are increased, colony formation is reduced, and proliferation is reduced [278]. Similarly, when expressed in MCF-7 breast cancer cells, GlialCAM increases cell motility and adhesion, decreases proliferation, and induces p53-mediated cellular senescence [279,283]. GlialCAM inhibits proliferation and β-catenin signalling in bladder carcinoma cells [284,285]. Furthermore, in renal carcinoma cells, GlialCAM decreases proliferation, induces cell cycle arrest, and stimulates c-Myc degradation [286]. GlialCAM expression is also sufficient for reducing Notch-mediated invasion and migration in prostate cancer cells [282]. Lastly, GlialCAM stabilises connexin-43 at cell-cell gap junctions [287], connexin-43 being a potential tumour suppressor itself [288,289]. In summary, GlialCAM has a strong anti-proliferative influence when expressed in cancer cells, which could underpin its role as a tumour suppressor.

### 5.2 Ca^2+^-sensitive Cl^−^ channels

Four single membrane-pass auxiliary subunits of CaCCs have been identified (known as Ca^2+^-activated Cl^−^ channel regulator or Cl^−^ channel accessory [CLCA]1-4) [290,291]. Interestingly, the molecular identities of the conducting subunits were only discovered later and termed Best1-4 and TMEM16 [[292], [293], [294], [295]]. CaCCs demonstrate voltage-dependence at steady-state, which is abolished following an increase in [Ca^2+^]_i_ [296]. Increased [Ca^2+^]_i_ also increases I_Cl_ and accelerates current onset [296]. CaCCs are expressed in epithelia and excitable tissues, where they regulate excitability [297], smooth muscle contraction [298] and fluid secretion [299]. Expression of CLCA1 and CLCA2 in HEK293 cells induces an enlarged and outwardly-rectifying I_CaCC_ [290,300]. More recent work has demonstrated that the secreted N-terminus of CLCA1, produced following autoproteolysis, is sufficient to stabilise TMEM16 A at the membrane, increasing I_CaCC_ [[301], [302], [303]]. CLCA1 contains an intrinsic metalloprotease domain in the N-terminus that is thought to be responsible for autoproteolysis and regulating mucus turnover in the colon [304]. Despite CLCA2 enlarging I_CaCC_, CLCA2 does not interact directly with TMEM16 or Best1 [305]. Instead, CLCA2 interacts directly with store-operated Ca^2+^ channels, Orai1 and STIM-1, stimulating ER Ca^2+^ replenishment following cytosolic depletion [305].

CLCAs have a well-documented tumour-suppressive role [[306], [307], [308]]. CLCA1 is downregulated in colorectal and pancreatic cancer specimens [306,[309], [310], [311]]. CLCA1 knockdown induces proliferation and inhibits differentiation of caco-2 colorectal cancer cells [311]. Furthermore, CLCA1 overexpression inhibits Wnt signalling and colorectal tumour growth and metastasis *in vivo* [306]. CLCA2 expression is also decreased in high-grade nasopharyngeal, colorectal, lymphoid and breast cancer specimens compared to low grade samples [307,[312], [313], [314]]. Expression of CLCA2 decreases nasopharyngeal and breast tumourigenesis *in vivo* [307,312,315]. Similarly, CLCA2 depletion increases the number of circulating prostate tumour cells in mice [316]. At a cellular level, CLCA2 inhibits Wnt signalling [317], decreases invasion [315], inhibits proliferation [312], induces transcellular adhesion [316], inhibits epithelial-to-mesenchymal transition [312,316], induces differentiation [316,318], inhibits focal adhesion kinase [312,319] and induces p53-mediated cellular senescence [320]. The ability of CLCA2 to inhibit cancer cell migration appears to be I_Cl_ independent, as inhibiting I_Cl_ has a further anti-migratory effect in cells expressing CLCA2 as well as having an anti-migratory effect in cells not expressing CLCA2 [312]. Ramena et al. observed CLCA2 at cell-cell junctions, interacting with EVA1/ZO-1 or β-catenin [317]. Sequestration of β-catenin at the plasma membrane was therefore suggested as a mechanism for CLCA2-induced inhibition of epithelial-to-mesenchymal transition. CLCA4 expression is decreased in bladder, hepatocellular and breast cancer specimens compared to adjacent normal tissue [308,321,322]. CLCA4 expression also decreases tumourigenicity in mice [321]. Furthermore, CLCA4 depletion induces epithelial-to-mesenchymal transition via PI3K/Akt signalling [308,322]. Despite the abundance of evidence implicating CLCAs as tumour suppressor genes, CLCAs have also been implicated in induction of lung colonization *in vivo* via adhesive interactions between endothelial CLCA and β_4_ integrin expressed on circulating cancer cells [323,324]. Similarly, increased CLCA2 expression is seen in circulating lung adenocarcinoma cells and ovarian cancer cell aggregates [325,326], suggesting CLCAs may potentially be tumour suppressors on the one hand, and metastasis-promoting on the other.

## 6 Conclusion

**Fig. 5:**
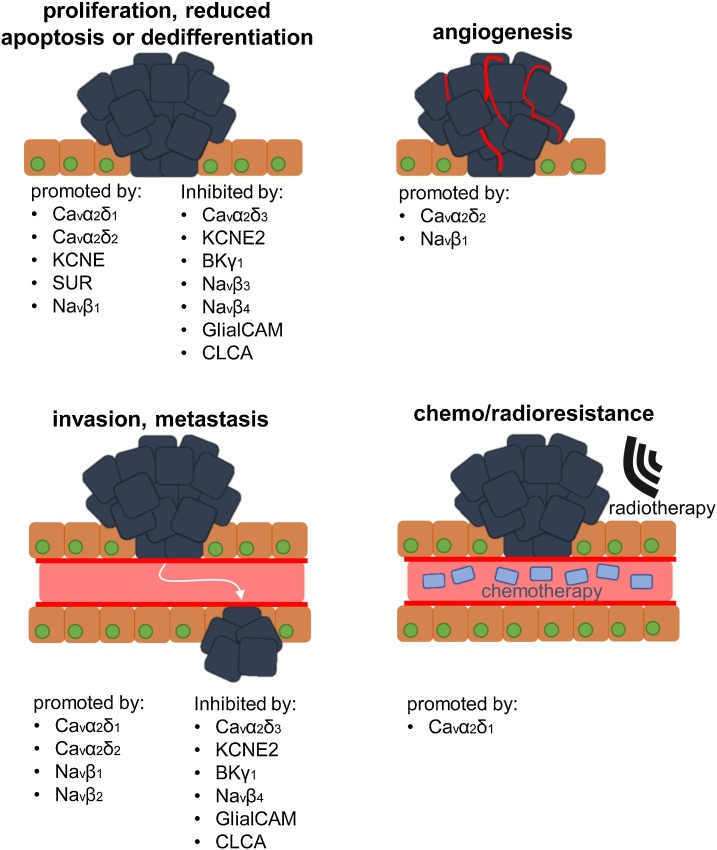
Involvement of ion channel auxiliary subunits in different stages of tumour progression. A number of different ion channel auxiliary subunits are up- or down-regulated in cancer cells promoting proliferation, reducing apoptosis and differentiation. Other auxiliary subunits have been shown to regulate angiogenesis, invasion, and metastasis, thus promoting tumour progression. Finally, ion channel auxiliary subunits may also play a role in chemo/radioresistance, underscoring the potential importance of these proteins in relation to therapeutic intervention.

Many ion channel auxiliary subunits are upregulated, e.g. Ca_v_βs, or downregulated, e.g. K_v_βs, in tumours and thus may represent novel cancer biomarkers. *in vitro* and *in vivo* experimentation has further implicated various auxiliary subunits in tumour formation and progression, such as Na_v_β_1_ and α_2_δ_1_ (Fig. 5). However, others, e.g. CLCAs, Na_V_β_3/4_, may function as tumour suppressors. Clearly, it is important from a treatment perspective to understand the mechanistic function of ion channel auxiliary subunits, including the extent that they contribute to cancer progression through potentiating ion conductance or via non-conducting signalling. For example, α_2_δ_1_- and α_2_δ_2_-induced Ca^2+^ influx may promote hepatocellular carcinoma cell sphere formation and pancreatic adenoma proliferation respectively [71,83]. Other examples include Na_V_α-dependent, Na_V_β_1_-mediated process outgrowth and the extent of glibenaclamide-induced inhibition of SUR2-mediated cancer cell proliferation correlating with the mRNA expression of Kir6.2 [213,249]. Validating the contribution of ion conductance to the oncogenic function of these auxiliary subunits would provide a potential therapeutic target, as many ion channel inhibitors are already in clinical use and could be repurposed [[327], [328], [329]]. On the other hand, numerous auxiliary subunits many regulate cancer progression via non-conducting roles, e.g. regulation of transcription, proliferation and differentiation by Ca_v_β_1_ and KChIP3 [36,172]. Various auxiliary subunits also function as adhesion molecules in cancer cells, e.g. GlialCAM, CLCAs and Na_v_βs [254,278,316]. Further work is required to fully delineate the diverse functional contributions of these subunits to carcinogenesis, tumour progression and metastasis, and understand their potential as novel therapeutic targets.

## Conflicts of interest statement

The authors declare that they have no conflicts of interest.
